# Improving Health Care Transition for Young Patients With Sickle Cell Disease Through Quality Network

**DOI:** 10.1001/jamanetworkopen.2025.4957

**Published:** 2025-04-15

**Authors:** Ofelia A. Alvarez, Carrie Gann, Debbie Ringdahl, Manisha Bansal, Farranaz Alvarez Nunez, William Slayton, Reema Kashif, Tung Wynn, Elizabeth Bradley, Kathryn Diener, Katrina Rivers, Sherry Buchman, Angela Chatfield, Monica Heldreth, Sandra Widland, Liz Y. Bayes, Sue Butts-Dion

**Affiliations:** 1 Division of Pediatric Hematology-Oncology, University of Miami, Miami, Florida; 2 Johns Hopkins All Children’s Hospital, St Petersburg, Florida; 3 University of Florida, Gainesville; 4 Nemours Health, Jacksonville, Florida; 5 Orlando Health Arnold Palmer, Orlando, Florida; 6 Now with Pfizer, Rare Disease Division, Orlando, Florida; 7 Florida Department of Health, Tallahassee; 8 National Institute for Children’s Health Quality, Boston, Massachusetts; 9 Department of Pediatrics, University of Miami, Miami, Florida

## Abstract

**Importance:**

Transitioning from pediatric to adult health care is crucial for the continuity of care for young adults with sickle cell disease. Among 5 pediatric hematology practices participating in the Florida Pediatric Hematology Learning and Action Network, 3 practices lacked transition programs before this quality improvement project.

**Objective:**

To evaluate the implementation of programs to improve care for individuals transitioning from pediatric to adult care for sickle cell disease.

**Design, Setting, and Participants:**

This multicenter observational quality improvement study was conducted at 5 pediatric hematology centers in Florida. All patients with sickle cell disease ages 14 to 21 years without neurocognitive impairment who attended 1 of the 5 centers over the project period were included. Data were analyzed from June 2022 to December 2023.

**Exposure:**

The network participated in virtual learning about quality improvement and the Got Transition health care transition framework. Clinics organized transition programs and used the Model for Improvement and plan-do-study-act cycles. Readiness assessments and transition plans were implemented. Data updates were conducted every month. P-charts were created to present aggregate and individual practice improvement.

**Main Outcomes and Measures:**

Percentage of patients with sickle cell disease ages 14 to 21 years attending a clinic at each center with a readiness assessment and a transition plan at least once during the previous 12 months as verified by monthly electronic health record (EHR) audits.

**Results:**

Among 627 patients who met criteria for transition implementation because of age and diagnosis, 458 patients were assessed (240 male [52.4%] and 218 female [47.6%]). Collective change initiatives included educating the clinical staff about the health care transition framework, establishing processes for identifying eligible patients using the patient roster, and creating reliable processes and procedures to ensure that patients had a readiness assessment and transition plan. Over the 19-month project, monthly EHR reviews of 100% or a sample of 10 random EHRs demonstrated an increase in the aggregate mean percentage of eligible patients with readiness assessment from 68.8% (95% CI, 63.1%-74.5%) to 87.8% (95% CI, 85.6%-89.5%), representing a 27.6% improvement. The aggregate transition plan increased from 58.8% (95% CI, 53.3%-64.2%) to 81.2% (95% CI, 78.9%-83.5%), with sustained metrics for over 12-month-period.

**Conclusions and Relevance:**

These findings suggest that collaboration among centers was associated with successful achievement of goals. Educating and sharing responsibilities among all center clinicians, identifying patients ahead of time through EHR reviews and clinic-staff huddles, and having electronic versions of readiness assessment and dot phrases to document plans were associated with sustained outcomes.

## Key Points

**Question:**

Can a state quality improvement network organize and implement sickle cell transition programs from pediatrics to adult care?

**Findings:**

In this quality improvement study of 458 pediatric patients with sickle cell disease, 5 hematology centers successfully implemented health care transition programs. More than 80% of eligible patients had readiness assessments and transition plans assigned within a 19-month period.

**Meaning:**

This study found that education and sharing responsibility among clinicians, identifying patients ahead of time, and having electronic versions of transition documents were associated with sustained processes.

## Teaser

This quality improvement study investigates the use of a quality improvement network to address health care transition from pediatrics to adult care among youths with sickle cell disease.

## Introduction

A sampling of youths and families from the 2016 National Survey of Children’s Health demonstrated that fewer than 20% were counseled for health care transition.^1^ As with other chronic illnesses, sickle cell disease (SCD) presents adolescents and young adults with many challenges when they transition from pediatric to adult-centered health care.^2,3^ These challenges involve the disease itself, which often worsens during late adolescence, with higher health care use and mortality,^4,5,6^ stressors affecting personal decision-making without the same parental protection or support, frequent health insurance changes or health insurance loss,^7^ and lack of knowledge to navigate the health care system, among other biopsychosocial factors. It is well documented that the transition years between ages 19 and 24 years are associated with higher mortality rates for people living with SCD.^5,6^ Having adequate transition preparation may increase patient integration into adult practice in diabetes^8,9^ and SCD^10^ programs and is valued by clinicians^11^ and patients.^12^ It is likely that better continuity of care could ameliorate morbidity and mortality.^13^

Recognizing these challenges, the Florida Children’s Medical Services (CMS) and the National Institute for Children’s Health Quality (NICHQ) have supported the Florida Pediatric Hematology Learning and Action Network (LAN), consisting of 5 pediatric hematology-oncology programs throughout Florida, to engage in a state quality improvement (QI) project centered on SCD transition from pediatric to adult care. NICHQ supported the initiative by offering organizational structure and QI education. CMS provided partial funding for the salaries of medical clinicians and nurse practitioners but did not pay for transition coordinators.

There were 2 sites that had previously participated in the Patient-Centered Outcomes Research Institute–sponsored clinical trial The Sickle Cell Trevor Thompson Transition Project (ST3P Up),^13^ which helped to develop or grow SCD pediatric and adult transition programs from 2019 to 2022 and tested whether the addition of virtual peer mentoring was superior to education alone. ST3P Up trained 14 participating sites during 4 years of QI learning and coached them to successfully apply the Six Core Elements of the Got Transition health care transition framework.^14^ There were 3 centers that did not have an established transition program and did not participate in ST3P Up.

Our state project aimed to spread knowledge, experience, and best practices to centers without established pediatric transition programs and to further improve existing programs with the guidance of state leadership and national experts in QI. It differed from ST3P Up in that it focused on QI without paying for a transition coordinator and working only with program-available resources. Additionally, virtual peer mentoring was unavailable. Our project processes were aligned with sustainable practice settings.

## Methods

This quality improvement study was deemed exempt from further review after its classification as a quality improvement initiative by the University of Miami Institutional Review Board. Informed consent was waived given that this was a quality improvement project that did not directly affect patient care and no personal identifying information was required. The study is reported following the Standards for Quality Improvement Reporting Excellence (SQUIRE) 2.0 reporting guideline.

### Definitions and Quality Improvement Framework

Health care transition is defined as the purposeful, planned movement of adolescents and young adults with chronic physical and medical conditions from child-centered to adult-oriented health care systems.^14^ The Model for Improvement was used as the QI framework,^15^ with the health care transition framework serving as the model for transitioning from pediatric to adult care.^14^ The Six Core Elements of Health Care Transition framework are transition policy, patient tracking, readiness assessment (RA), transition plan (TP), transfer, and adult practice integration. During the previous 2 years, our centers engaged in 2 QI projects: transition policy development with patient input (core element 1) and identification and education of clinicians treating adult patients who could accept transitioned patients. This project focused on core elements 3 (RA) and 4 (TP) which are pivotal for developing effective transition programs.

### SMART Aim Statement

This project involved a specific, measurable, achievable, relevant, and time-bound (SMART) aim statement. Under this statement, by December 2023, 80% or more of patients ages 14 to 21 years with SCD attending a clinic at 1 of the 5 hematology centers would have a RA and TP updated at least once during the previous 12 months as assessed on monthly electronic health record (EHR) audits.

### Florida Pediatric Hematology Learning and Action Network

The LAN consists of 5 pediatric hematology oncology practices in Florida: the University of Miami, University of Florida, Nemours Jacksonville, Orlando Health Arnold Palmer, and Johns Hopkins All Children’s Hospital. There are 8 physicians (including O.A.A., M.B., F.A.N., W.S., R.K., T.W., and L.Y.B.), 2 nurse practitioners (C.G. and D.R.), 2 nurses (E.B. and K.D.), and 4 community advocates who actively participate in the network. The Table details the population and administrative capacity of the LAN. The investigators observed race (Black, White, multiracial, and other) and ethnicity (Hispanic and non-Hispanic) for reporting purposes. Florida CMS is the administrative sponsor arranging for monthly meetings and following up on QI outcomes. The CMS leadership consists entirely of nurses. NICHQ is the organization that consults and teaches QI methodology.

**Table.** Population and Administrative Capacity of Learning Action Network

Characteristic	Site A	Site B	Site C	Site D	Site E
University affiliated	No	Yes	No	Yes	Yes
Site urbanicity	Urban	Urban	Urban	Urban	Suburban
Patients with SCD served, No.	363	325	283	404	350
Patients eligible for transition based on age and cognition, %^a^	116 (31.9)	106 (32.6)	120 (42.4)	145 (35.9)	140 (40.0)
Representative sample size analyzed from patients seen in clinic during previous month	10 Random EHRs	10 Random EHRs; 100% EHRs reviewed during last 6 mo	10 Random EHRs	100% EHRs	10 random EHRs
EHR system	Epic	Epic	Epic	Epic	Epic
Staff actively involved in QI project	1 Physician	1 Physician	1 Physician and 1 nurse	1 Nurse practitioner	1 Nurse practitioner
Other contributing staff^b^	1 Nurse practitioner and 3 hematology nurses	2 Nurse practitioners	1 Physician and 1 hematology nurse practitioner and two hematology nurses	1 Physician, 1 hematology nurse, and 1 social worker	2 Nurse practitioners and 1 physician
Transition champion	Physician	Physician	Physician	Nurse practitioner	Nurse practitioner

Abbreviations: EHR, electronic health record; QI, quality improvement; SCD, sickle cell disease.

^a^ Patient eligibility for transition of care was based on patient lists for sites A through D and on an estimate for site E.

^b^ Staff who helped with the readiness assessment and plan administration but may not have actively participated in learning action network educational activities and virtual meetings.

### Interventions and Change Ideas

Change ideas were developed collectively by the group or tailored to a specific program based on program-level decisions. These ideas were then tested using plan-do-study-act cycles ^16^ Collective change ideas consisted of clinical staff education about the health care transition framework and QI strategies, identification of eligible patients through the patient roster, development of a standard way of measuring progress, and distribution of process ownership among staff members to ensure sustainability. Program-specific change ideas were a prospective review of patients who were scheduled to come to the clinic within the week, clinic-staff huddles to discuss patients eligible for transition, the option of converting the single-page American Society of Hematology (ASH) SCD transition RA^17^ or 7-page Transition Intervention Program–Readiness for Transition (TIP-RFT) SCD RA^18^ into EHR format, and the use of dot phrases to facilitate documentation of the RA and plan in the EHR.

### Inclusion and Exclusion Criteria

Patients were eligible for the transition process if they were aged 14 to 21 years, had SCD of any genotype, came to participating practices during the project period, and were neurocognitively capable of completing an RA in English by themselves and follow a TP as judged by the practice staff. Formal neurocognitive testing was not necessary for exclusion.

### Outcome Measures

We selected 2 outcome measures: the percentage of patients with a RA every month, derived from the number of patients with a current RA seen in clinic the previous month (numerator) divided by all eligible patients who came to clinic in the previous month (denominator), and the percentage of patients with a TP, derived from the number of patients with a current plan seen in the clinic the previous month (numerator) divided by all eligible patients who came to the clinic in the previous month (denominator). The rationale for choosing these measures was to track progress that would address the SMART aim. The denominator included only patients who were seen given that we did not have control over which patients did not follow up.

### Staff Training and Work Assignment

Virtual education sessions provided by NICHQ lasted 3 hours every 3 months. There was 1 office hour offered every month for coaching. Data and learning from QI strategies and plan-do-study-act cycles were reviewed twice a month with all LAN members in virtual 1-hour meetings conducted by CMS leadership. When progress was not made, sessions allowed for discussion of what was working well for some centers and what could possibly work well for others. There were 2 QI consultants who encouraged and coached sites. Center personnel who were involved shared the information with other people within their center to disseminate ideas and promote collaboration. No sites had a paid transition coordinator, although generally 1 person became the champion to oversee processes. Therefore, there were no added expenses to the intervention.

### Data Collection and Tracking

Epic EHR review of patients with SCD who were eligible by age, were not impaired neurocognitively, and attended clinic during the previous month allowed for easy identification. A designated staff member identified patients who had completed outcome measures at each site. We selected 10 EHRs at random from the total using an online random number generator, and the staff reviewed whether elements were met or not. Because the absence of outcome measures was a rare event for sites B and D, they opted to review 100% of EHRs all the time or during the last part of the project to detect changes in process performance. Data were entered monthly into the NICHQ website, documenting breakthroughs, tests, lessons learned, and challenges faced. Having a spreadsheet database for tracking transition elements was encouraged but not required.

### Statistical Analysis

Means and medians calculated for individual sites and as aggregate data for the percentage of RAs and TPs were shown in tables during monthly virtual meetings. Initially, run charts were automatically produced by the NICHQ team and posted on the website to analyze data due to an insufficient number of data points for Shewhart charts (P-charts). Run charts allowed teams to visualize data trends and shifts over time, indicating outcomes associated with tested changes. The median of the first 3 data points served as the baseline. The team updated the baseline median as improvement signals emerged using QI macros.^19^ Once 19 points were available, the data were transferred to a Shewhart chart to distinguish between common (random) and special (nonrandom) variation. We identified special cause variation and shifted the mean centerline when 8 or more consecutive data points consistently fell above the centerline after an intervention. Teams annotated their charts to track changes and analyze contributions of these changes to the improvement, guiding further improvement actions. We used the χ^2^ test to calculate *P* values and determine statistical significance between baseline and intervention periods, as well as before and after mean centerline adjustment. Statistical significance was set at *P* < .05, and all tests were 2-sided.The analysis was conducted using SAS statistical software version 9.4 (SAS Institute). Data were analyzed from June 2022 to December 2023.

## Results

### Initial Steps of the Intervention

From June 2022 to December 2023, a total of 627 eligible patients met criteria to join the transition program, among whom 486 patients (77.5%) were eligible at the start of the project and 458 patients (73.0% of the total) came to clinic and were assessed (240 male [52.4%] and 218 female [47.6%]; 434 non-Hispanic African American or Black [94.8%] and 24 Hispanic with African American or Black, White, or multiple races [5.2%]). There were 285 patients (62.2%) with hemoglobin SS, 135 patients (29.5%) with hemoglobin SC, 22 patients (4.8%) with sickle ß^+^ thalassemia, and 16 patients (3.5%) with sickle ß^0^ thalassemia. We excluded 6 patients clinically: 1 patient with cerebral palsy, 1 patient with severe Down syndrome, and 4 patients with a history of stroke and an inability to perform independent assessment. We also excluded 2 adolescents and young adults for autism confirmed by neurocognitive testing. Our baseline data points included June through August 2022, serving as a surrogate baseline due to the absence of prior data for the new process.^20^ For outcome measures, the baseline mean aggregated percentage of RAs and TPs across all sites was 64.0% (95% CI, 57.2%-70.9%) and 51.3% (95% CI, 44.2%-58.5%), respectively, reflecting outcomes associated with 2 sites that had organized programs.

### RA and TP Implementation

During 2 to 8 weeks, each center leadership educated clinic staff about the QI project. Each site used a different method to track patients, including a spreadsheet database (Excel version 2502 [Microsoft]) and review of medical records ahead of time. Once eligible patients were identified, the physician or nurse practitioner informed patients about the importance of completing the RA before or after their medical evaluation but during the same clinical encounter. The clinician reviewed patient responses with the patient and formulated a plan, taking into consideration patient responses that he or she did not know and wanted to learn. At that time, targeted education was provided, and frequently a task was assigned to be completed by the time of the next clinic encounter. In addition, sites C and D developed a specific curriculum to cover the most important topics of transition, including disease knowledge, how to navigate the health care system, and learning about patient health insurance. Knowledge was tested with before and after questionnaires at site D.

### Evolution of Transition Performance for Individual Sites Over Time

**Figure 1.**
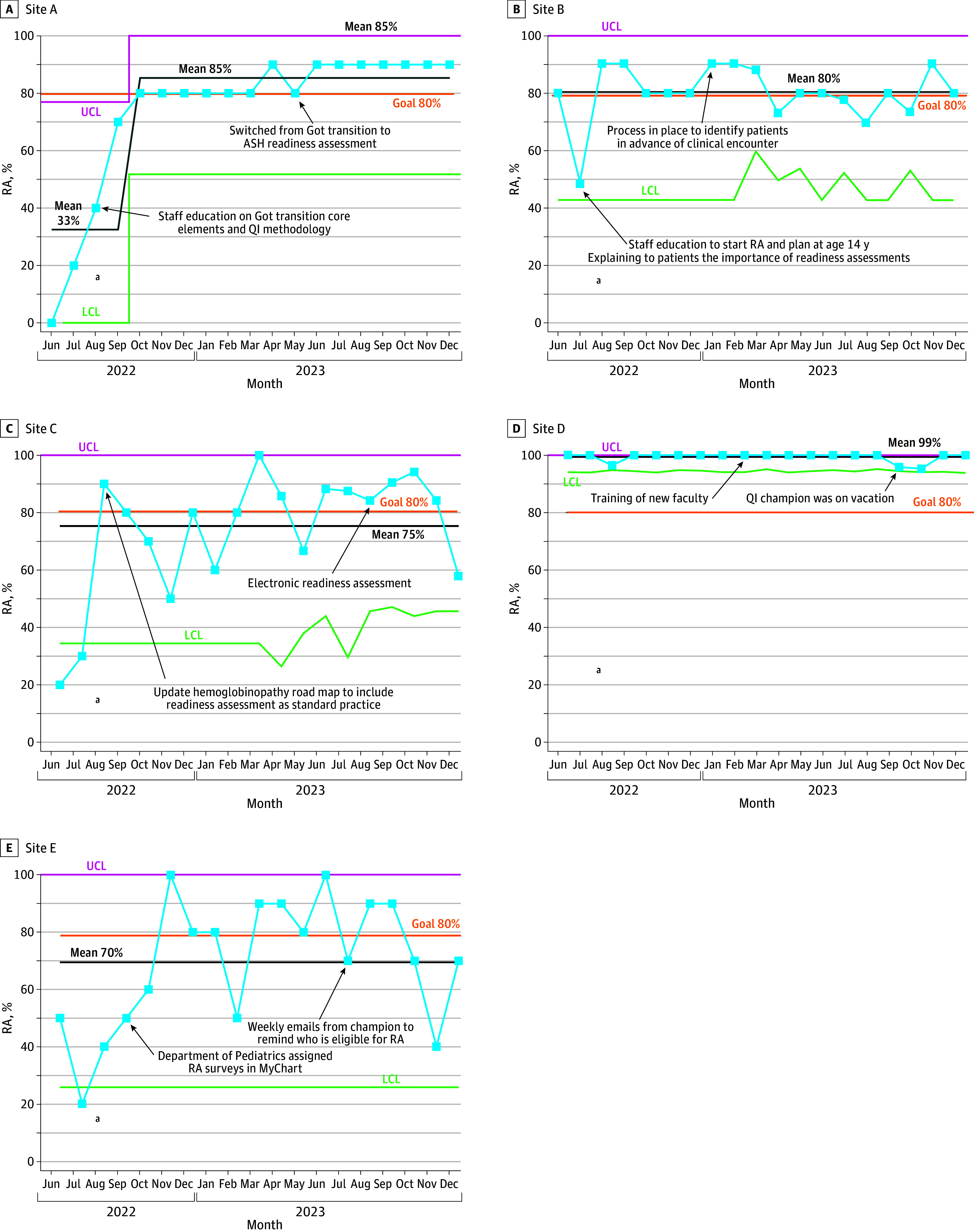
Readiness Assessment (RA) Statistical Control P-Charts for 5 Sites ASH indicates American Society of Hematology; LCL, lower control limit; QI, quality improvement; UCL, upper control limit. ^a^Denotes the start of the sitewide intervention (August to September 2022).

**Figure 2.**
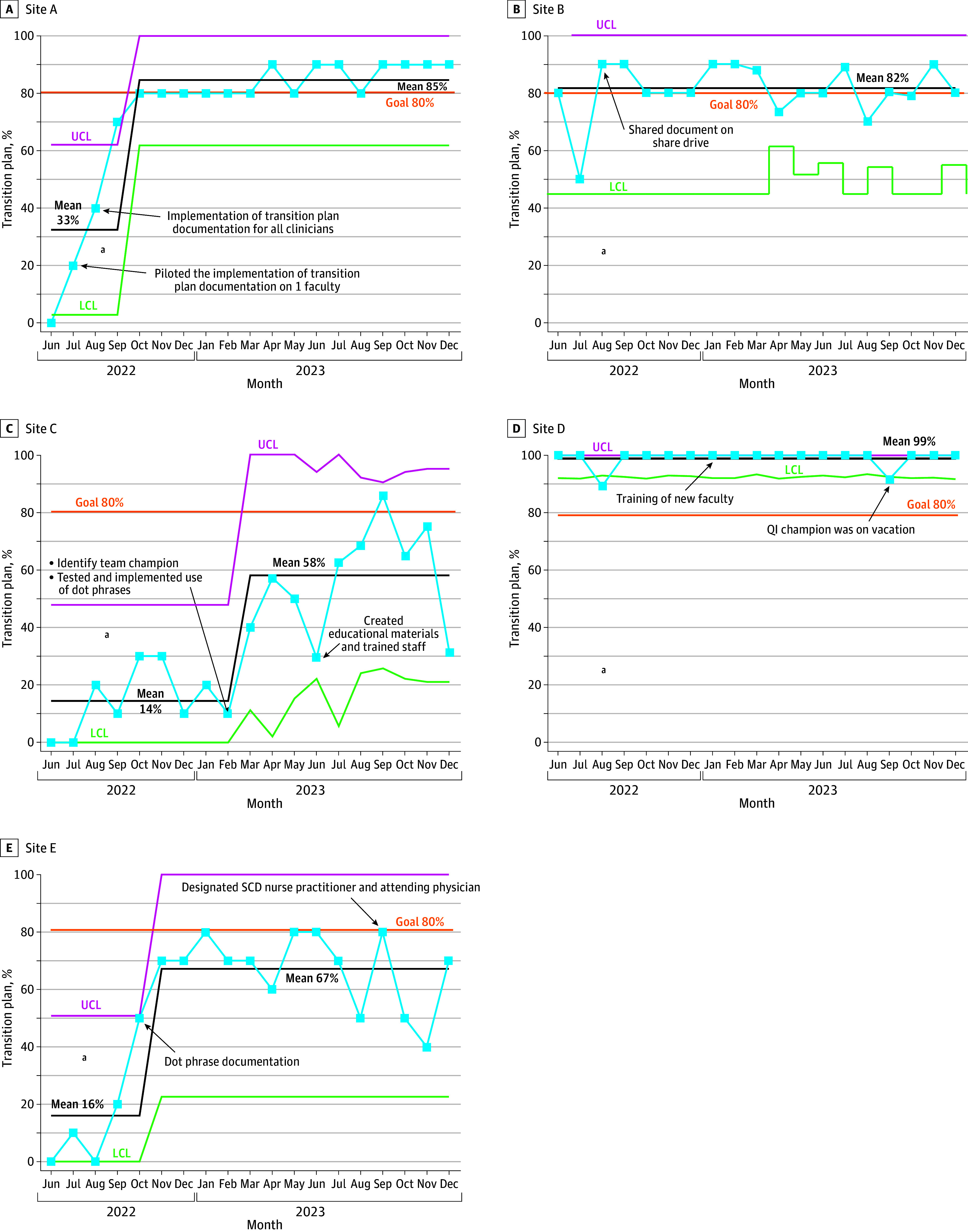
Transition Plan Statistical Control P-Charts for 5 Sites LCL, lower control limit; QI, quality improvement; SCD, sickle cell disease; UCL, upper control limit. ^a^Denotes the start of the sitewide intervention (August to September 2022).

Figure 1 and Figure 2 show control charts with intervention annotations in implementing RAs and TPs at the individual site level. Sites B and D had previously established SCD transition programs, and sites A, C, and E were new to adopt programs. There were no missing data.

Examining data for each site with data displayed monthly (Figure 1) revealed an increase in the percentage of RAs from baseline for all sites. Site A, B, and D achieved a mean centerline above 80%. For sites C and E, the mean centerline for RA was kept above 75% and 69%, respectively. Site B used a paper format for RA and TP, targeting patients’ lack of knowledge to formulate a plan. Site D used an electronic format for the same core elements and developed a transition curriculum. Site A rapidly implemented changes after learning what worked from other sites. Initially, site A used the health care transition framework RA and later adopted the ASH RA in August 2023.

Regarding individualized site performance for TP documentation in the EHR, sites A, B, and D stabilized their mean centerlines above our target of 80% by the end of the study period (Figure 2). Although sites C and E did not reach the target aim, they improved during the study period. Site C shifted the mean centerline from 14% to 58%, representing a 314% improvement, by March 2023. Site E shifted the mean centerline from 16 to 67, representing a 318% improvement. Sites C and E had a prolonged lag time in the adoption of EHR-based clinical support tools, with improvement in plan documentation when using dot phrases.

### Aggregate Outcome Data

**Figure 3.**
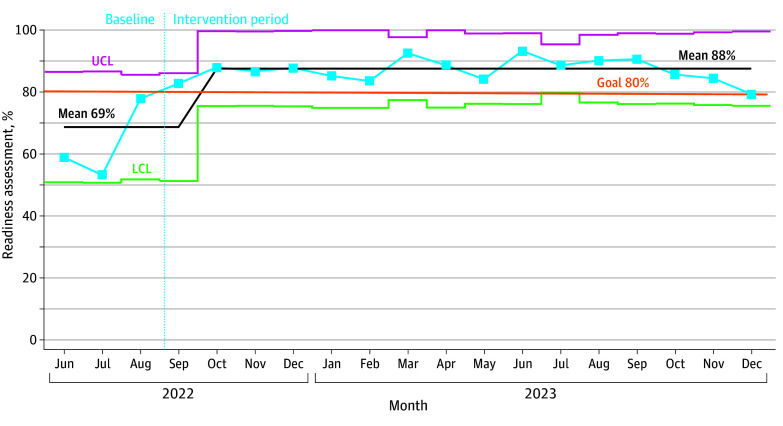
Statistical Control P-Chart of Aggregate Readiness Assessment Core Element LCL indicates lower control limit; UCL, upper control limit.

**Figure 4.**
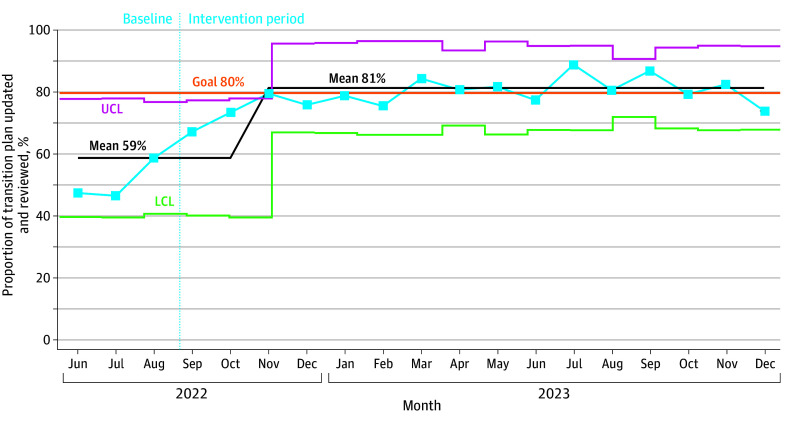
Statistical Control P-Chart of Aggregate Transition Plan Core Element LCL indicates lower control limit; UCL, upper control limit.

For aggregate outcome measures, the mean RA centerline was adjusted in October 2022 from 68.8% (95% CI, 63.1%-74.5%) to 87.8% (95% CI, 85.6%-89.5%; *P* < .001), representing a 27.6% improvement. We sustained this result over a 15-month period (Figure 3). For the aggregated percentage of TPs, the mean centerline was shifted in November 2022 from 58.8% (95% CI, 53.3%-64.2%) to 81.2% (95% CI, 78.9%-83.5%; *P* < .001), representing a 38.1% improvement that was sustained over a 13-month period (Figure 4).

### Problems With QI Project

Despite overall success, challenges included time constraints for staff in busy clinics and staff turnover, necessitating continuous training for new personnel. Sometimes, patients were seen by physicians or nurse practitioners who were not fully aware of the QI project, with an associated decrease in the percentage for that month. That was the case for site B in July 2022, when the SCD doctor was on vacation, with a low astronomical point for this period. At site E, there was a change in program leadership toward the end of the project, with temporarily decreased performance. It was also noted that the implementation of EHR-based assessments could take months to develop.

## Discussion

### Summary of Findings

In this quality improvement study, our network was successful in working together and sharing knowledge. As discussed, 3 of 5 programs did not have an established SCD transition program at onset. Sharing knowledge with those programs was associated with rapid capacity building. We met the aggregate goal of 80% of patients having current RAs and TPs, with rapid improvement over a 6-month period. Some individual sites are continuing to work on the goal. There was no added monetary cost to the clinics in implementing the programs, which is important for the project to be easily applicable to other programs. However, the QI project required having committed and organized clinicians to assume new roles and responsibilities.

There were several program and process differences between the ST3P Up clinical trial^21^ and our QI project regarding the RA and TP: (1) 4 of our sites used the briefer tool, the ASH SCD transition RA, instead of the longer TIP-RFT; (2) we did not have a transition coordinator; (3) our QI was supported by the state rather than a research funder; and (4) we concentrated on RA and TP, whereas ST3P Up used a self-scored and validated process-measurement tool based on all transition core elements to assess progress. Their aggregate RA percentage at baseline was 38%, achieving a median shift by 6 months to 76%. TP percentages were not collected or analyzed in ST3P Up. Therefore, our baseline aggregate percentage for both core elements was higher because 2 of our sites had already participated in ST3P Up.

### Interpretation

In discussion, our group found 3 best QI practices. First was educating and sharing responsibility among all clinicians (clear roles and expectations), while also maintaining a champion or person leading the project; this was critical to maintain performance even when the champion was on vacation or otherwise away. Second was identifying patients ahead of time through preparatory EHR review or huddles. Third, having an electronic version of the RA and documenting plans with dot phrases on the EHR helped to sustain the program once implemented, facilitating documentation. The QI activity led to the creation and enhancement of transition programs at the 5 centers, including the development of patient databases to streamline the transition process and measure outcomes. Site D, which had an established educational curriculum, automatization through EHR, and previous training with ST3P-Up, had outstanding program sustainment.

### Limitations

This study has several limitations, 2 of which were time constraint to perform the work in a busy clinic and changes of personnel over time, which required training in the project. For site E, including the RA within the EHR was a challenge, taking 3 months to implement. Another limitation is that this QI project was focused on core elements 3 and 4 without exploring transfer of care (core element 5) or integration to adult care (core element 6). Therefore, the efficacy of the intervention for patient outcomes cannot be fully appreciated.

## Conclusions

Findings from this quality improvement study suggest that cooperation among centers to improve health care quality is feasible and could serve as a model for similar initiatives. We plan to sustain these QI processes by implementing an EHR checklist tracking tool and a transition pop-up reminder as our next QI projects and eventually study the integration of patients into the adult practice. We also plan to measure the impact of the process on patients by assessing the confidence they experience with health care transition using the same RA questions. Having a successful program eventually may translate into excellent integration to the adult practice after a satisfactory transition process, aiming for the lowest morbidity possible and hopefully mitigating the higher mortality rate known to occur in this age group.

## Notes

Supplement.
Data Sharing Statement
