# Supplementary material for: Improving Health Care Transition for Young Patients With Sickle Cell Disease Through Quality Network
Source: JAMA Netw Open. 2025 Apr 15;8(4):e254957. doi: 10.1001/jamanetworkopen.2025.4957 (PMC12000992; doi:10.1001/jamanetworkopen.2025.4957)
Supplement: Supplement. — Data Sharing Statement [file jamanetwopen-e254957-s001.pdf]

Data Sharing Statement

Alvarez. Improving Health Care Transition for Young Patients With Sickle Cell Disease Through Quality Network. JAMA Netw Open. Published online April 15, 2025.
doi:10.1001/jamanetworkopen.2025.4957

Data
Data available: Yes
Data types: Deidentified participant data
How to access data: The data could be accessed by emailing corresponding author at oalvarez2@med.miami.edu
When available: With publication

Supporting Documents
Document types: None

Additional Information
Who can access the data: Researchers whose proposed use of the data has been approved.
Types of analyses: For a specified purpose.
Mechanisms of data availability: After approval of a proposal.
